# Europium Nanoparticle-Based Lateral Flow Strip Biosensors for the Detection of Quinoxaline Antibiotics and Their Main Metabolites in Fish Feeds and Tissues

**DOI:** 10.3390/bios14060292

**Published:** 2024-06-04

**Authors:** Qing Mei, Biao Ma, Yun Fang, Yunfei Gong, Jiali Li, Mingzhou Zhang

**Affiliations:** 1 Zhejiang Provincial Key Laboratory of Biometrology and Inspection & Quarantine, China Jiliang University, Xueyuan Street, Xiasha Higher Education Zone, Hangzhou 310018, China; 17854301575@163.com (Q.M.); 16a0701109@cjlu.edu.cn (B.M.); gongyf@cjlu.edu.cn (Y.G.); 2 Qianjiang Customs of the People’s Republic of China, Hangzhou 310012, China; shirly13867903555@163.com; 3 Hangzhou Quickgene Sci-Tech. Co., Ltd., Hangzhou 310018, China; qjc1993@126.com

**Keywords:** lateral flow strip biosensors, quinoxaline antibiotics, fluorescent nanomaterials, multiple detection

## Abstract

Olaquindox (OLA) and quinocetone (QCT) have been prohibited in aquatic products due to their significant toxicity and side effects. In this study, rapid and visual europium nanoparticle (EuNP)-based lateral flow strip biosensors (LFSBs) were developed for the simultaneous quantitative detection of OLA, QCT, and 3-methyl-quinoxaline-2-carboxylic acid (MQCA) in fish feed and tissue. The EuNP-LFSBs enabled sensitive detection for OLA, QCT, and MQCA with a limit of detection of 0.067, 0.017, and 0.099 ng/mL (R^2^ ≥ 0.9776) within 10 min. The average recovery of the EuNP-LFSBs was 95.13%, and relative standard deviations were below 9.38%. The method was verified by high-performance liquid chromatography (HPLC), and the test results were consistent. Therefore, the proposed LFSBs serve as a powerful tool to monitor quinoxalines in fish feeds and their residues in fish tissues.

## 1. Introduction

Olaquindox (OLA, N-(2-hydroxyethyl)-3-methyl-2-quinoxaline carboxamide-1,4-dioxide) and quinocetone (QCT, 3-methyl-2-quinoxalinbenzenevinylketo-1,4-dioxide) belong to quinoxaline antibiotics [1]. They have been widely used as medicinal feed additives and growth promoters in aquaculture to treat bacterial illnesses [2]. OLA and QCT can be rapidly metabolized in vivo, and their primary metabolite is 3-methyl-quinoxaline-2-carboxylic acid (MQCA) [3]. Numerous studies have demonstrated that MQCA can exert carcinogenic, mutagenic, and teratogenic effects on people [4]. The potential toxic residues of MQCA in fish tissue can affect human health [5]. Therefore, the use of OLA as a growth promoter was banned in the European Union in 1999 [6]. In China, OLA was banned for use in aquaculture [7,8]. Although tightly controlled, it is still broadly implemented in some regions as a feed additive for aquatic products because of its economic benefits in modern aquaculture [9]. Therefore, developing a portable, sensitive, and reliable analytical method to strengthen daily monitoring of OLA, QCT, and MQCA is necessary.

Presently, instrumental methods for quinoxaline antibiotics and their main metabolite include high-performance liquid chromatography (HPLC) and liquid chromatography-tandem mass spectrometry (LC-MS/MS) [10,11]. These methods possess high accuracy, recovery, reproducibility, and reliable quantitative analysis. However, these methods require intricate technical equipment and specialized technical operations. These processes are also expensive and can only be conducted in laboratories. In addition, time-resolved fluoroimmunoassay (TR-FIA) [12], dual-label direct competitive fluorescence-linked immunosorbent assay (dc-FLISA) [13], and enzyme-linked immunosorbent assay (ELISA) [14] have been successfully developed for detecting antibiotics in feeds and veterinary residues in animal tissues. Compared with instrumental methods, these methods have high-throughput detection capabilities, can analyze a large number of samples quickly, and are relatively simple to operate without sophisticated instrumentation. However, the tedious washing steps hinder the application of on-site testing [15]. Lateral flow strip biosensors (LFSBs) are highly efficient biosensors and can be used to detect antibiotics in a rapid, simple, specific, sensitive, and reliable way. They also allow the visualization and quantification of target products [16,17].

LFSBs commonly use gold nanoparticles (AuNPs) as labels. The method is simple and convenient, and results can be observed visually, but low sensitivity and a narrow linear range are considered drawbacks of AuNP-LFSBs [18]. Therefore, different fluorescence microspheres are employed for LFSB assays, such as quantum dots (QDs) [19], upconversion nanoparticles (UCNPs) [20], and europium nanoparticles (EuNPs) [21]. As the excitation and emission wavelengths of most fluorescent markers are in the ultraviolet-visible (UV-vis) wavelength range, membrane supports and biological components generate high fluorescence background interference due to light scattering and autofluorescence [22,23]. Their high fluorescence background diminishes the advantage of the strong signal intensity contributed by these fluorescent markers [24]. However, QDs are susceptible to photothermal effects [25]. Additionally, UNCPs have relatively low conversion efficiency and higher costs [26]. EuNP is a unique fluorescence material that can avoid background fluorescence from biological samples. As a result, EuNP is beneficial for improving sensitivity and accuracy while being less affected by the sample matrix [27]. EuNP-LFSB assays have shown great potential in large screening and point-of-care testing. According to the literature, EuNP-LFSB assays have been developed to detect clinical diagnosis [28], veterinary drug residues [29,30], and zoonotic strain assays [31].

This study established competitive EuNP-LFSBs for the rapid quantitative determination of OLA, QCT, and MQCA with high sensitivity. This is a reliable and instrument-free method for analyzing multiple analytes in a single sample. Furthermore, it advances the application of EuNP-LFSB assays in food safety and quality monitoring.

## 2. Materials and Methods

### 2.1. Reagents and Apparatus

3-dimethylaminopropyl-3-ethylcarbodiimide (EDC), N-hydroxysuccinimide (NHS), and 2-(N-Morpholino) ethanesulfonic acid (MES) were obtained from Sigma-A-drich (St. Louis, MO, USA). Carboxylate-modified EuNPs with a diameter of 200 nm were procured from Shanghai Uni Biotech Ltd. (Shanghai, China).OLA, olaquindox-ovalbumin (OLA-OVA), antiolaquindox monoclonal antibody (anti-OLA-mAb), QCT, quinocetone-ovalbumin (QCT-OVA), MQCA, 3-methyl-quinoxaline-2-carboxylic acid-bovine albumin (MQCA-BSA), goat antimouse IgG (GAM-IgG), carbadox, mequindox, cyadox, cinoquidox, drazidox, oxytetracycline, and Tween-20 were purchased from Hangzhou QuickGene Biotech Co., Ltd. (Hangzhou, China). Nitrocellulose (NC) membranes, sample pads, conjugate pads, and absorbent pads were obtained from Zhejiang Dien Biotech Corp (Hangzhou, China).

The XYZ3000 dispensing platform and CM2000 guillotine cutter (BioDot, Irvine, CA, USA) were used to prepare and cut test strips. The Fourier-transform infrared spectroscopy (FTIR) optics (ALPHA spectrometer, Bruker Optics GmbH, Ettlingen, Germany) was used to judge the coupling efficiency between EuNP and specific antibodies by the characteristic peaks of N–H (3500–3100 cm^−1^) in the amide bond. The FIC-S2011-B14 fluorescent strip reader (Suzhou, China) was used to read and record the fluorometric test strip assay. The touch-spin centrifuge (Mini-6KS, Allsheng Instruments Co., Ltd., Hangzhou, China) played a role in sample preparation. The LFSB results were validated by comparison with data obtained from an Agilent 1100 HPLC system (Agilent Tech, Santa Clara, CA, USA).

### 2.2. Preparation of EuNP-Labeled OLA-mAb

EuNP-mAb probes were prepared based on our previous work with slight modifications [32]. The operation was as follows: 5 mg of carboxylic EuNP, 2 mg of EDC, and 3 mg of NHS were added to 10 mL of MES (0.1 mol/L, pH 5.5). Then, the solution was incubated for 30 min with moderate shaking. Excess EDC was removed by centrifugation at 14,000 rpm/min for 15 min. Additionally, 2 mL phosphate buffer solution (PBS, 0.01 M, pH 7.4) was added to the precipitates and mixed thoroughly. Then, anti-OLA-mAb (10, 20, 40, and 60 μg/mL) was added and incubated at room temperature for 2 h. For inhibiting the nonspecific locus, 500 μL 1% BSA (*w*/*v*) was added to the aforementioned solution. The diluent of BSA was PBS (0.01 M, pH 7.4). After 30 min, the solution was centrifuged twice at 14,000 rpm/min for 15 min to wash off excess BSA. Finally, the precipitate was dissolved in 2 mL of BBS buffer solution (0.05 M, pH 8.0, 1% (*w*/*v*) BSA, and 0.5% (*v*/*v*) Tween 20) and stored at 4 °C for further use. Then, the above samples were tested by FTIR optics. Each sample was scanned thrice, and the working spectral range was 400–4500 cm^−1^. The preparation of EuNP-QCT-mAb and EuNP-MQCA-mAb probes was consistent with the above methods.

### 2.3. Preparation of AuNP-mAb Probe

According to the sodium citrate reduction method used in the previous experiment [2], AuNPs were prepared with chloroauric acid in the presence of a reducing agent of sodium citrate and coupled with mAbs (10 μg/mL).

### 2.4. Fabrication of EuNP-LFSBs

This LFSB system has five parts: a sample pad, conjugate pad, NC membrane, absorption pad, and backing card. Sample pads were immersed in PBS buffer solution (0.05 M, pH 7.4, containing 2% (*w*/*v*) sucrose, 0.5% (*w*/*v*) BSA, and 0.5% (*v*/*v*) Tween-20), dried at 37 °C for 2 h. EuNP-mAbs were sprayed on the binding pad and then placed in a desiccator at 37 °C for 2 h. OLA-OVA, QCT-OVA, and MQCA-BSA were spotted on the NC membrane as the test line (T line), and GAM-IgG was spotted on the NC membrane as the control line (C line). They were dried at 37 °C for 2 h. The sample pad, NC membrane, and absorption pad were attached to the backing card, cut into 2.5-mm test strips, and stored at room temperature for spare use. For EuNP-LFSBs, a total of 40 μL of the sample solution was dropped onto the sample pad. After 10 min of reaction, the findings were visualized under a portable 365 nm UV light. Simultaneously, the EuNP-LFSBs were inserted into the fluorescent strip reader to record the fluorescence intensity on the T and C lines. Subsequently, the fluorescence intensity was plugged into the respective standard curves to determine the analyte concentrations.

### 2.5. Assessment of Specificity and Sensitivity

The sensitivity of the LFSBs was determined using the limit of detection (LOD) as a quantitative parameter. To obtain the LOD of the EuNP-LFSBs, a mixed standard solution was diluted to a range of 0.01–50 ng/mL, and a competitive inhibition curve was obtained for the inhibition rate (1 − B/B0) × 100% against the analyte’s logarithmic concentration. The positive control (B) and negative control (B0) were calculated by the ratio of the fluorescence intensity of T and C lines (FI_T_/FI_C_), respectively. The linear range of this analysis was set to the concentration that aroused 10–80% inhibition.

The specificity of the LFSBs was assessed by detecting standards (50 ng/mL) containing only one or more target antibiotics and observing whether the 3 test lines produced cross results. To further evaluate the specificity of the LFSBs, other quinoxaline antibiotics were tested, including carbadox, mequindox, cyadox, cinoquidox, drazidox, and oxytetracycline.

### 2.6. Precision

The precision of this LFSB system was assessed by the intra-batch and inter-batch experiments. Five LFSBs from the same batch were selected and tested with negative samples to complete the batch experiment. Inter-batch experiments were completed by testing five LFSBs from different batches. Each LFSB was scanned thrice. The coefficient of variation between batches was measured by recording T/C values.

### 2.7. Analysis of Spiked Samples—Recovery Assessment

Fish feeds and tissue samples were brought from a local supermarket, and all samples were certified as negative by HPLC. Then, samples were spiked with different concentrations of target substances. The concentrations of QCT, OLA, and MQCA were 0.2, 0.4, and 0.6 ng/mL, respectively. The three standards were added individually and mixed in the spiking experiment. Each concentration was repeated thrice. The average recovery rate and relative standard deviation (RSD) were calculated for accuracy.

The fish tissue sample preparation process was as follows [33]. Briefly, 2 g of fish was placed in a centrifuge tube, and then 6 mL of acetonitrile was added to the tube and vigorously mixed for 5 min. After, the supernatant was collected by centrifugation (8000 rpm/min for 10 min). Finally, the extract was dried and dissolved in PBS.

The fish feed sample preparation process was as follows [11]. Briefly, 10 mL of 5% aqueous methanol was added in 1 g of fish feed, oscillated at a constant temperature for 45 min, and the supernatant was collected by centrifugation at 3500 rpm/min for 10 min.

### 2.8. Analysis of Actual Samples

Five hundred actual samples were acquired from a local market, including 250 fish tissues and 250 fish feeds. All purchased fish tissues were frozen. These samples were analyzed using the EuNP-LFSB and HPLC assays.

Actual samples were tested by HPLC, referring to a previous experimental protocol [34]. Briefly, the actual samples were detected using an aqueous MilliQ solution of 0.08% acetic acid, methanol, acetonitrile (gradient elution), and a Zorbax Eclipse XDB C18 column. The flow rate was 0.6 mL/min, and the injection volume was 40 μL. The column temperature was 25 °C. The column temperature was 0.5 μg/kg.

### 2.9. Data Analysis

The schematic diagram was drawn using Photoshop (Photoshop CS3, Adobe Systems, San Jose, CA, USA). Graphs such as standard curves were plotted using Origin 9.0 (Origin Lab, Northampton, MA, USA). The data were organized using Microsoft Excel (Excel 2010, Microsoft Corporation, Redmond, WA, USA).

## 3. Results and Discussion

### 3.1. Detection Principle

**Figure 1:**
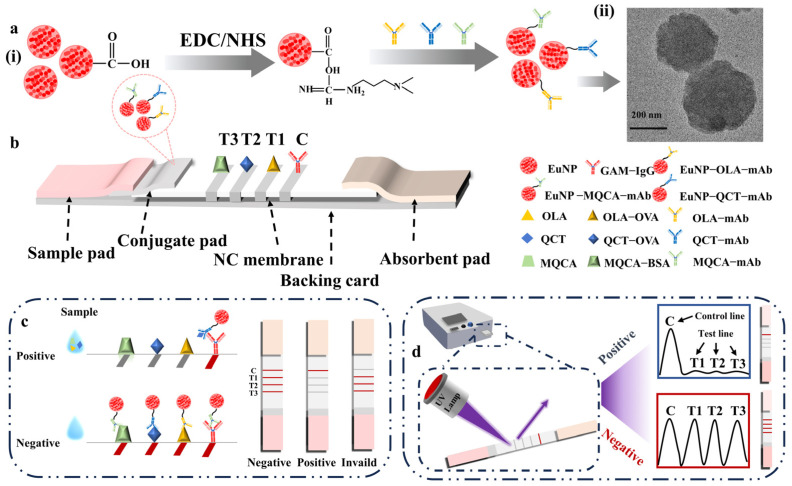
Detection principle of EuNP-LFSBs. (**a**(**i**)) Preparation of three EuNP-mAb probes. The EuNP-mAb probes were obtained by the activated ester method. (**a**(**ii**)) Scanning electron microscope images of EuNP-mAbs. (**b**) Composition of the EuNP-LFSB system. The fabrication of EuNP-LFSBs includes five parts: a sample pad, conjugate pad, NC membrane, absorption pad, and backing card. (**c**) The EuNP-LFSB detection schematic. The negative results for OLA, QCT, and MQCA were indicated by the appearance of test line 1 (T1), test line 2 (T2), and test line 3 (T3), respectively, and the control line (C) on the LFSBs. On the contrary, the positive result is indicated by the absence of test lines. (**d**) Visual identification and quantitative analysis of EuNP-LFSBs. The results were observed by naked eyes under UV light and the fluorescence intensity on the EuNP-LFSBs was read and stored by a fluorescent strip reader.

The detection principle of EuNP-LFSBs was based on the competitive reaction [35]. The schematic diagram of the EuNP-LFSBs is shown in Figure 1. A total of 40 µL of detection sample was added dropwise to the sample pad. The solution flowed to the other side by capillary force. If the solution contained the target substance, the target analyte would compete with the coating antigen on the NC membrane to bind to the EuNP-labeled-mAb. Therefore, the number of EuNP-mAb probes captured by the T line decreased, and a drop in fluorescence intensity indicated a positive result. In contrast, if the target analyte were absent in the solution, the EuNP-mAb probe would be captured by the T line and generate a fluorescent detection line visible under UV light. This phenomenon indicated a negative result. Whether the sample was negative or positive, the C line exhibited a fluorescence intensity. After the reaction, the fluorescence signals of the T and C lines were collected and analyzed by a fluorescence reader.

### 3.2. Characterization of EuNP-mAbs Probes

**Figure 2:**
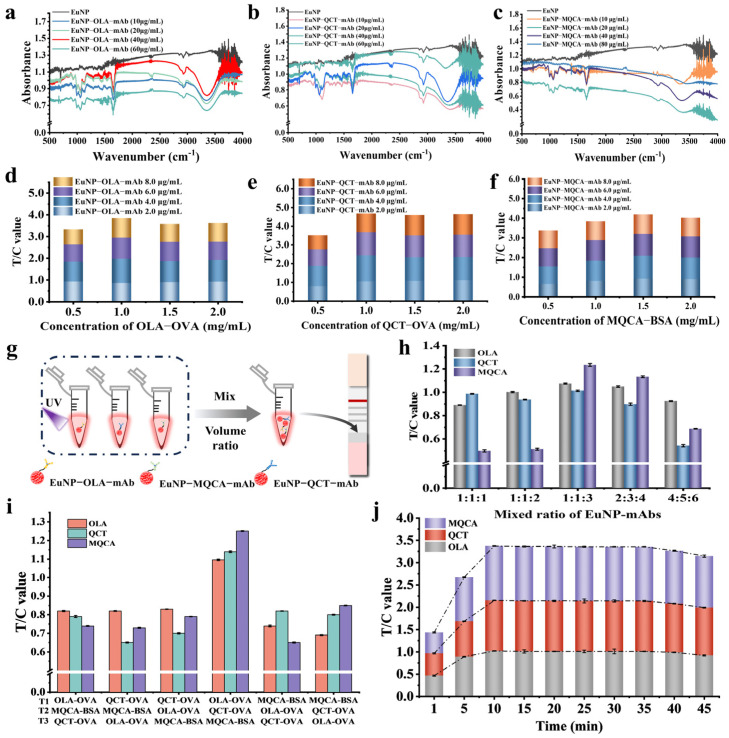
Optimization of the EuNP-LFSB system. (**a**) FTIR results for OLA. (**b**) FTIR results for QCT. (**c**) FTIR results for MQCA. (**d**) Influence of various OLA-OVA and EuNP-OLA-mAb concentrations on the T/C value. (**e**) Influence of various QCT-OVA and EuNP-QCT-mAb concentrations on the T/C value. (**f**) Influence of various MQCA-BSA and EuNP-MQCA-mAb concentrations on the T/C value. (**g**) Schematic diagram of three types of mixed probe preparation. (**h**) Effect of different EuNP-mAb probes mixed ratio on the fluorescence intensity. (**i**) Position result of the three encapsulated antigens on EuNP-LFSBs. (**j**) Effect of reaction time on EuNP-LFSBs.

Choosing the optimal amount of antibody labeling is beneficial for a more sensitive detection [36]. In this work, EuNPs modified with carboxylic groups were coupled with mAbs under the EDC/sulfo-NHS-mediated reaction. Furthermore, 10, 20, 40, and 60 μg/mL of the OLA-mAb, QCT-mAb, and MQCA-mAb were used for the preparation of EuNP-OLA-mAb, EuNP-QCT-mAb, and EuNP-MQCA-mAb probes, respectively. The coupling efficiency between EuNP and specific antibodies was judged by the characteristic peaks of N–H (3500–3100 cm^−1^) in the amide bonds. The results of FTIR are shown in Figure 2. We observed an absorption peak at 3500–3100 cm^−1^, indicating the presence of amide bonds. Among them, the maximum absorption peaks presented at 40 μg/mL for OLA-mAb and MQCA-mAb and at 20 μg/mL for QCT-mAb (Figure 2a–c). Then, by detecting the fluorescence intensity, the concentrations of OLA-mAb, QCT-mAb, and MQCA-mAb were finally chosen at 40, 20, and 40 μg/mL (Figure S1a–c), respectively.

### 3.3. Optimization of the EuNP-LFSB Parameter

Various factors were optimized to obtain the best signal intensity, including the concentrations of coating antigen and EuNP-mAbs on the binding pad, the mixed ratio of three EuNP-mAb probes, the best combination position of the three analytes, and the immunoreaction time.

The appropriate coating concentration provides enough binding sites to allow the target material to bind effectively to the test material and form a stable complex. The appropriate EuNP-mAb probe concentration ensures that the labeled signal’s intensity is within the detection system’s response range for accurate quantification. This study optimized the concentrations of OLA-OVA, EuNP-OLA-mAb, QCT-OVA, EuNP-QCT-mAb, MQCA-BSA, and EuNP-MQCA-mAb. The concentrations of EuNP-OLA-mAb were 2.0, 4.0, 6.0, and 8.0 μg/mL and that of OLA-OVA were 0.5, 1.0, 1.5, and 2.0 mg/mL. Similar parameter optimizations were performed for QCT and MQCA. According to the best signal intensity and T/C value, the concentration of OLA-OVA was 1.0 mg/mL and that of EuNP-OLA-mAb was 4.0 μg/mL (Figure 2d). The concentrations of QCT-OVA and EuNP-QCT-mAb were consistent with that of OLA-OVA and EuNP-OLA-mAb (Figure 2e). The best result was obtained when the concentrations of MQCA-BSA and EuNP-MQCA-mAb were 1.5 mg/mL and 4.0 μg/mL, respectively (Figure 2f).

Based on the above experiments, the optimal concentrations of the three fluorescent probes and the three coating antigens were selected. To achieve the purpose of detecting three targets simultaneously, three EuNP-mAb probes were mixed with the volume ratios of 1:1:1, 1:1:2, 1:1:3, 2:3:4, and 4:5:6 (Figure 2g). According to the fluorescence intensity, we selected the optimal volume ratios of probes and then detected the color rendering intensity of the three coating antigens in three different binding areas on the NC membrane. According to Figure 2h, the best ratio for the EuNP-mAb probes was 1:1:3, determined by the fluorescence intensity and T/C value.

By placing the coated antigen in the right place, it can be sufficiently exposed and bound to the target molecule in the sample to be tested, thereby increasing the assay’s sensitivity. In addition, the correct placement of the coating antigen can also reduce the occurrence of nonspecific binding or cross-reactivity, increasing the assay’s specificity. In order to obtain the best location of the binding area, the color rendering intensity of OLA, QCT, and MQCA were tested in three different binding areas based on the optimization results. The best results were obtained when OLA-OVA was in the T1 position, QCT-OVA was in the T2 binding region, and MQCA-BSA was in the T3 binding region (Figure 2i).

Optimization of reaction time can increase the ability of LFSB detection systems to capture and recognize target molecules, thereby improving detection sensitivity. In the study, 10 different time points (1, 5, 10, 15, 20, 25, 30, 35, 40, and 45 min) were used to obtain the optimal reaction time. The results are shown in Figure 2j. The T/C value showed a sharp trend of change with the increase of reaction time in the first 5 min and stabilized and reached equilibrium within 10 min. In order to meet the needs of on-site inspection, 10 min was selected as the best reaction time.

### 3.4. Optimization of the AuNP-LFSB Parameter

A similar parameter optimization was performed for the AuNPs-LFSB system, and the result of parameter optimization for AuNP-LFSBs was shown in Supplementary Material Figure S4.

### 3.5. Sensitivity and Specificity

**Figure 3:**
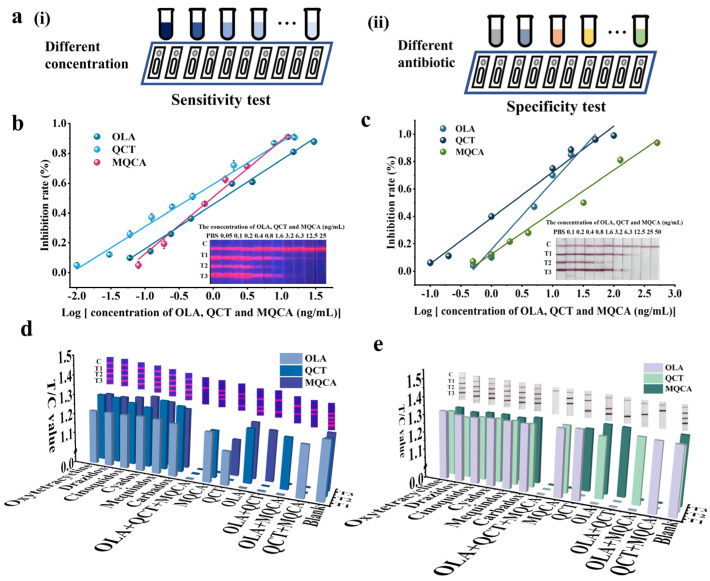
EuNP-LFSB performance evaluation. (**a**(**i**)) Schematic diagram of the sensitivity test. (**a**(**ii**)) Schematic diagram of the specificity test. (**b**) Sensitivity analysis of EuNP-LFSBs. The OLA standard curve was y = 0.3005x + 0.4548, R^2^ = 0.9906. The QCT standard curve was y = 0.2837x + 0.6035, R^2^ = 0.9924. The MQCA standard curve was y = 0.4036x + 0.5045, R^2^ = 0.9914. (**c**) Sensitivity analysis of AuNP-LFSBs. The OLA standard curve was y = 0.5211x + 0.1582, R^2^ = 0.9803. The QCT standard curve was y = 0.3367x + 0.386, R^2^ = 0.9884; The MQCA standard curve was y = 0.2981x + 0.1243, R² = 0.9847. (**d**) Specificity analysis of EuNP-LFSBs. (**e**) Specificity analysis of AuNPs-LFSBs.

In this study, a series of diluents ranging from 0.01 to 50 ng/mL in a PBS solution were prepared and detected by EuNP-LFSBs, repeating three times for each concentration (Figure 3a(i)). Then, the standard curves for each OLA, QCT, and MQCA were established. For EuNP-LFSBs, the LOD for OLA, QCT, and MQCA was 0.067, 0.017, and 0.099 ng/mL, respectively (Figure 3b). Compared to the published rapid detection method for OLA, QCT, and MQCA, the EuNP-LFSB method showed a higher sensitivity (Table 1). For AuNP-LFSBs, the LOD for OLA, QCT, and MQCA was 0.77, 0.14, and 0.83 ng/mL, respectively (Figure 3c). The published rapid detection methods could only be used on a single sample. The EuNP-LFSBs established in this study allow for the simultaneous detection of OLA, QCT, and MQCA.

The specificity of the LFSBs was assessed by detecting several commonly used quinoxaline antibiotics and the three standards were added individually or mixed together. Each analyte diluted to a standard solution with a final concentration of 50 ng/mL was tested by the developed LFSBs (Figure 3a(ii)). The cross-reactivity of EuNP-LFSBs and AuNP-LFSBs was less than 0.076% and 0.072%, respectively (Figure 3d,e). The results showed great specificity. By performing specific experiments, other interference factors of nonspecific binding were excluded to ensure the accuracy and reliability of the detection results.

### 3.6. Precision

The precision of the EuNP-LFSBs was estimated by intra-batch and inter-batch experiments. As shown in Figure S3a,b, the CV% of the intraassay was less than 3.18%, and the interassay was less than 6.26%, indicating the high repeatability of the system.

### 3.7. Detection of Spiked Samples by EuNP-LFSBs

To reflect the influence of the sample matrix, EuNP-LFSBs were checked by testing spiked blank samples with three repetitions for each concentration. From the results presented in Table 2, the average recoveries of OLA were 98.58–104.17% with RSD less than 9.38% when using the experimental spiked concentrations, the average recoveries of QCT were 98.25–102.69% with RSD less than 5.68%, and MQCA recoveries were 98.01–103.17% with an RSD less than 5.06%. The results indicated that this method is highly consistent and accurate. Despite the results remaining highly consistent, a slight error also existed. For example, the recovery rates for OLA, QCT, and MQCA were greater than 100%. The reason might be interfering substances in the sample, which competed with the target analyte, resulting in a higher recovery rate.

### 3.8. Detection of Real Samples by EuNP-LFSBs and HPLC

**Figure 4:**
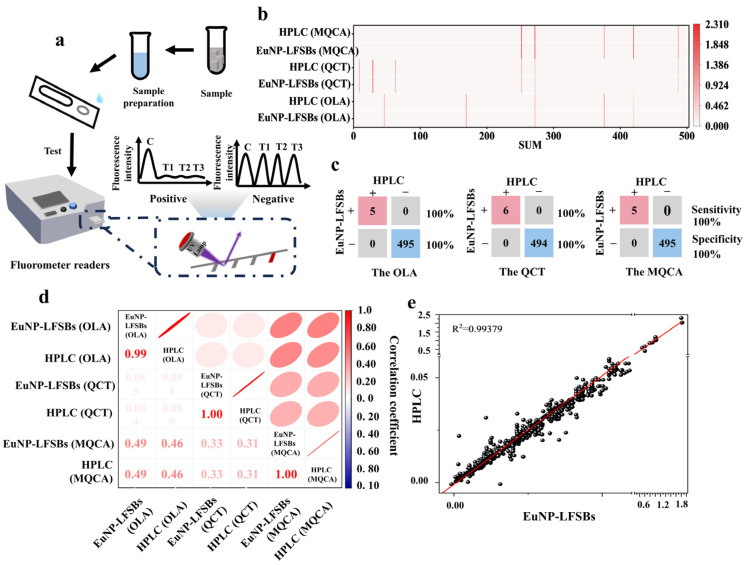
Testing in actual samples. (**a**) Schematic diagram of actual sample analysis. (**b**) The results of EuNP-LFSBs and HPLC for 500 actual samples. (**c**) Table of concordance between the proposed EuNP-LFSBs and HPLC for 500 actual samples tested (sensitivity values [%] used to detect EuNP-mLFIA are shown in red; specificity values [%] are shown in black). (**d**) The correlation analysis of EuNP-LFSBs and HPLC. (**e**) Comparison of the consistency of 500 actual samples detected by EuNP-LFSBs and HPLC.

To further verify the reliability of the EuNP-LFSBs, 500 actual samples were analyzed with EuNP-LFSBs and HPLC (numbers 1–250 were fish feeds; numbers 251–500 were fish tissues). To ensure the accuracy of the analytical results, each sample was analyzed thrice to compare the correlation between the two methods. The detection process of EuNP-LFSBs is shown in Figure 4a. The results of EuNP-LFSBs and HPLC for 500 actual samples were consistent (Figure 4b). The sensitivity and specificity were calculated to be 100% (Figure 4c). In addition, EuNP-LFSBs and HPLC were analyzed by correlation, and as shown in Figure 4d, the two methods were well correlated. The consistency of the two methods was compared with R^2^ > 0.99, indicating that EuNP-mLFIA correlated well with HPLC (Figure 4e).

## 4. Conclusions

EuNP-based competitive LFSBs were successfully established for multiresidue detection of the two quinoxaline antibiotics and their main metabolites in fish feeds and tissues. Analyses of OLA, QCT, and MQCA were completed in 10 min. The sensitivity of OLA, QCT, and MQCA of EuNP-LFSBs reached 0.067, 0.017, and 0.099 ng/mL, respectively. The sensitivity was increased by 9–11 times compared with AuNP-LFSBs. Both the established EuNP-LFSBs and AuNP-LFSBs have good specificity. The recoveries of the three target substances ranged from 98.01–104.17% in spiked sample experiments. The results of the actual samples were consistent with those of the HPLC method. Therefore, they exhibited high sensitivity, good accuracy, and satisfactory specificity. OLA and QCT were representative quinoxaline antibiotics, and other types might also be present in the same sample. More testing lines could be added to the EuNP-LFSBs to improve the detection of more target analytes. Furthermore, broad-spectrum antibodies could be developed to detect a class of target analytes, reducing analysis time and cost. In summary, the EuNP-LFSB method would be appropriate for detecting quinoxaline antibiotics and their main metabolites in fish feeds and tissues. This method could be extended to other small molecules to monitor food safety and environmental pollution.

## Figures and Tables

**Table 1:** Comparison of the published rapid detection method for OLA, QCT, and MQCA.

Method	Test Substance	Sample	LOD	Reference
CL-ciELISA	MQCA	Fish samples	0.01 μg/kg	[4]
UCNP-ICG	OLA	Fish muscle and water samples	1.42 ng/mL	[33]
AuNP-ICG	QCT	Animal feed	3.9 ng/mL	[18]
EuNP-LFSBs	OLA, QCT, MQCA	Fish feed and tissue	0.067, 0.017, 0.099 ng/mL	This work

CL-ciELISA: Competitive indirect enzyme-linked immunosorbent assay. UCNP-IA UCNP-ICG: Upconversion nanoparticle-based immunochromatographic. AuNP-ICG: Colloidal gold immunochromatographic.

**Table 2:** Recovery rate determination of OLA, QCT, and MQCA in spiked samples by EuNPs-mFIA analyses.

Sample	Spiked Level (ng/mL)			Detected Result (ng/mL)			Average Recovery Rate			RSD ^a^ (n = 3)		
	OLA	QCT	MQCA	OLA	QCT	MQCA	OLA	QCT	MQCA	OLA	QCT	MQCA
Fish feed	0.2	/	/	0.201 ± 0.004	/	/	100.67%	/	/	1.88%	/	/
	0.4	/	/	0.399 ± 0.007	/	/	99.58%	/	/	1.71%	/	/
	0.6	/	/	0.600 ± 0.040	/	/	100.22%	/	/	0.44%	/	/
	/	0.2	/	/	0.203 ± 0.006	/	/	100.77%	/	/	3.48%	/
	/	0.4	/	/	0.399 ± 0.003	/	/	99.75%	/	/	0.75%	/
	/	0.6	/	/	0.602 ± 0.213	/	/	100.42%	/	/	0.53%	/
	/	/	0.2	/	/	0.201 ± 0.005	/	/	99.92%	/	/	2.32%
	/	/	0.4	/	/	0.389 ± 0.019	/	/	98.17%	/	/	5.06%
	/	/	0.6	/	/	0.600 ± 0.005	/	/	99.78%	/	/	0.92%
	0.2	0.2	/	0.199 ± 0.003	0.197 ± 0.002	/	99.17%	98.25%	/	1.62%	1.11%	/
	0.4	0.4	/	0.398 ± 0.014	0.401 ± 0.003	/	99.67%	100.25%	/	3.18%	0.66%	/
	0.6	0.6	/	0.605 ± 0.040	0.599 ± 0.003	/	100.72%	99.67%	/	0.77%	0.58%	/
	0.2	/	0.2	0.199 ± 0.002	/	0.202 ± 0.005	99.25%	/	101.42%	1.31%	/	3.52%
	0.4	/	0.4	0.495 ± 0.007	/	0.403 ± 0.005	123.75%	/	100.83%	1.32%	/	1.25%
	0.6	/	0.6	0.600 ± 0.030	/	0.599 ± 0.048	99.97%	/	99.92%	0.69%	/	0.80%
	/	0.2	0.2	/	0.198 ± 0.003	0.201 ± 0.002	/	99.00%	100.77%	/	1.52%	1.26%
	/	0.4	0.4	/	0.402 ± 0.003	0.407 ± 0.01	/	99.42%	100.33%	/	2.58%	3.49%
	/	0.6	0.6	/	0.603 ± 0.005	0.602 ± 0.043	/	100.25%	100.22%	/	0.94%	0.75%
	0.2	0.2	0.2	0.204 ± 0.017	0.197 ± 0.002	0.196 ± 0.005	100.32%	98.25%	98.43%	9.38%	1.11%	2.44%
	0.4	0.4	0.4	0.495 ± 0.006	0.503 ± 0.001	0.501 ± 0.003	98.8%	102.4%	100.2%	0.40%	2.77%	0.53%
	0.6	0.6	0.6	0.604 ± 0.005	0.599 ± 0.035	0.601 ± 0.045	104.4%	99.5%	99.7%	0.56%	3.54%	4.74%
Carp	0.2	/	/	0.204 ± 0.017	/	/	100.32%	/	/	9.38%	/	/
	0.4	/	/	0.394 ± 0.005	/	/	98.58%	/	/	1.55%	/	/
	0.6	/	/	0.604 ± 0.001	/	/	100.61%	/	/	0.10%	/	/
	/	0.2	/	/	0.203 ± 0.007	/	/	100.50%	/	/	3.89%	/
	/	0.4	/	/	0.399 ± 0.003	/	/	99.67%	/	/	0.63%	/
	/	0.6	/	/	0.603 ± 0.201	/	/	100.22%	/	/	0.35%	/
	/	/	0.2	/	/	0.196 ± 0.005	/	/	98.43%	/	/	2.44%
	/	/	0.4	/	/	0.391 ± 0.011	/	/	98.33%	/	/	2.95%
	/	/	0.6	/	/	0.601 ± 0.196	/	/	100.22%	/	/	0.35%
	0.2	0.2	/	0.200 ± 0.012	0.200 ± 0.01	/	99.84%	101.67%	/	5.86%	5.68%	/
	0.4	0.4	/	0.405 ± 0.002	0.401 ± 0.003	/	101.25%	100.25%	/	0.49%	0.66%	/
	0.6	0.6	/	0.599 ± 0.003	0.599 ± 0.003	/	99.94%	99.67%	/	0.63%	0.58%	/
	0.2	/	0.1	0.195 ± 0.012	/	0.199 ± 0.005	97.59%	/	99.67%	7.80%	/	2.26%
	0.4	/	0.5	0.417 ± 0.015	/	0.401 ± 0.003	104.17%	/	100.25%	5.89%	/	0.66%
	0.6	/	1.0	0.598 ± 0.008	/	0.601 ± 0.005	99.50%	/	99.94%	1.66%	/	0.79%
	/	0.2	0.2	/	0.202 ± 0.009	0.201 ± 0.002	/	100.33%	100.67%	/	4.30%	1.03%
	/	0.4	0.4	/	0.415 ± 0.013	0.416 ± 0.011	/	102.69%	103.17%	/	3.49%	3.01%
	/	0.6	0.6	/	0.599 ± 0.003	0.599 ± 0.004	/	100.06%	99.78%	/	0.63%	0.68%
	0.2	0.2	0.2	0.201 ± 0.005	0.201 ± 0.005	0.199 ± 0.003	99.01%	99.02%	98.02%	4.97%	4.54%	2.56%
	0.4	0.4	0.4	0.404 ± 0.001	0.401 ± 0.003	0.391 ± 0.011	102.01%	100.20%	98.01%	4.75%	0.53%	2.35%
	0.6	0.6	0.6	0.504 ± 0.005	0.599 ± 0.030	0.601 ± 0.505	99.6%	100.06%	102.13%	3.80%	3.53%	2.05%

^a^ RSD: relative standard deviation. “/”: Not detected.

## Data Availability

The data presented in this study are available in the article.

## Supplementary Materials

The following supporting information can be downloaded at: https://www.mdpi.com/article/10.3390/bios14060292/s1, Figure S1: Optimization of the EuNP-LFSBs system. Figure S2: Stability results of the EuNP-LFSBs. Figure S3: Intraassay and Interassay testing of EuNP-LFSBs. Figure S4: Optimization of the AuNP-LFSBs system.
