# Supplementary material for: Europium Nanoparticle-Based Lateral Flow Strip Biosensors for the Detection of Quinoxaline Antibiotics and Their Main Metabolites in Fish Feeds and Tissues
Source: Biosensors (Basel). 2024 Jun 4;14(6):292. doi: 10.3390/bios14060292 (PMC11202277; doi:10.3390/bios14060292)
Supplement: Supplementary file 1 [file biosensors-14-00292-s001.zip › biosensors-3020494-supplementary.pdf]

Supplementary Materials

Optimization of the EuNP–LFSB system

In the study, various additional conditions were optimized including different pH, temperature, and humidity conditions. In order to obtain the best pH of the PBS buffer, the color rendering intensity of OLA, QCT, and MQCA were tested in different pH values, ranging from 5 to 9. For EuNP–LFSBs, when the pH value was 8, the corresponding EuNP–LFSB methods had the maximum T/C values (Figure S1e). Therefore, the PBS buffer was better adjusted to weak alkalinity. Different humidity conditions and temperature conditions were optimized to obtain LFSBs with stable performance. Based on the best signal intensity, T/C value, and variance analysis, the humidity conditions were 50% (Figure S1e), and the temperature conditions were 18–26 ℃ (Figure S1f). Therefore, the best results were obtained when the humidity of the room was 50% at standard room temperature (18–26°C).

Stability of the EuNP–LFSB system

To detect the stability of the LFSBs, we put the test strips in an oven at 50 ℃ for 21 days for an accelerated destruction stability test. As could be seen from Figure S3, the T/C value almost did not change. The CV of OLA, QCT, and MQCA was 1.53%, 1.27%, and 1.18%. It demonstrated that the newly EuNP–LFSBs were stable and may be stored at room temperature for up to one year.

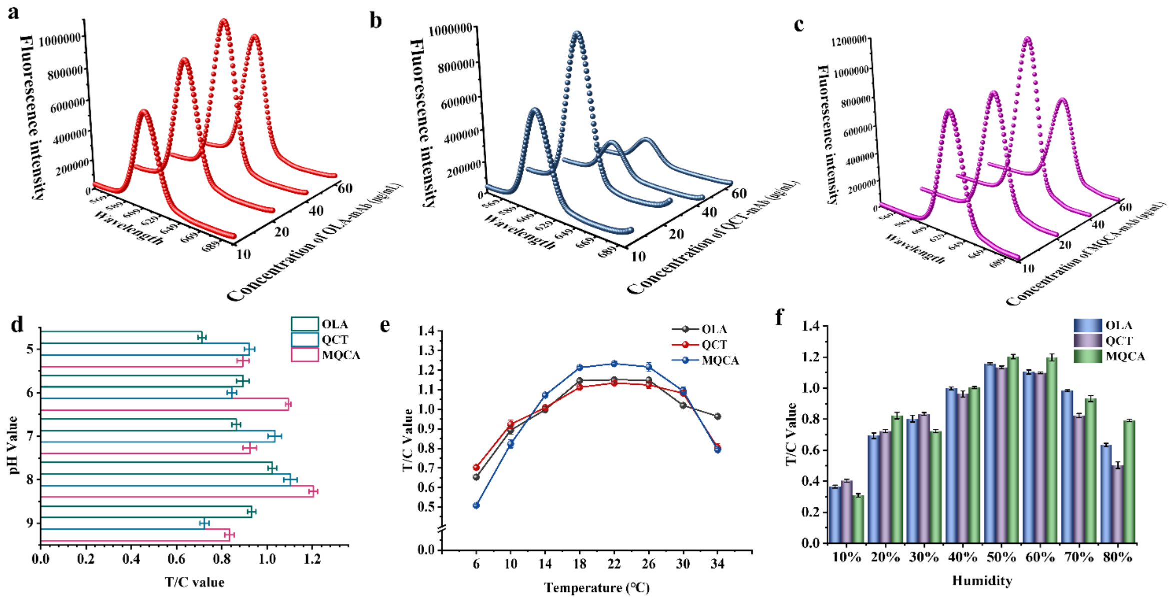

Figure S1. Optimization of the EuNP–LFSB system. (a) Effect of different OLA–mAb concentrations on the fluorescence intensity of the EuNP–OLA–mAb probe. (b) Effect of different QCT–mAb concentrations on the fluorescence intensity of the EuNP–QCT–mAb probe. (c) Effect of different MQCA–mAb concentrations on the fluorescence intensity of the EuNP–MQCA–mAb probe. (d) The effect of various pH levels on eEuNP–LFSBs. (e) The effect of humidity on EuNP–LFSBs. (f) The effect of temperature on EuNP–LFSBs.

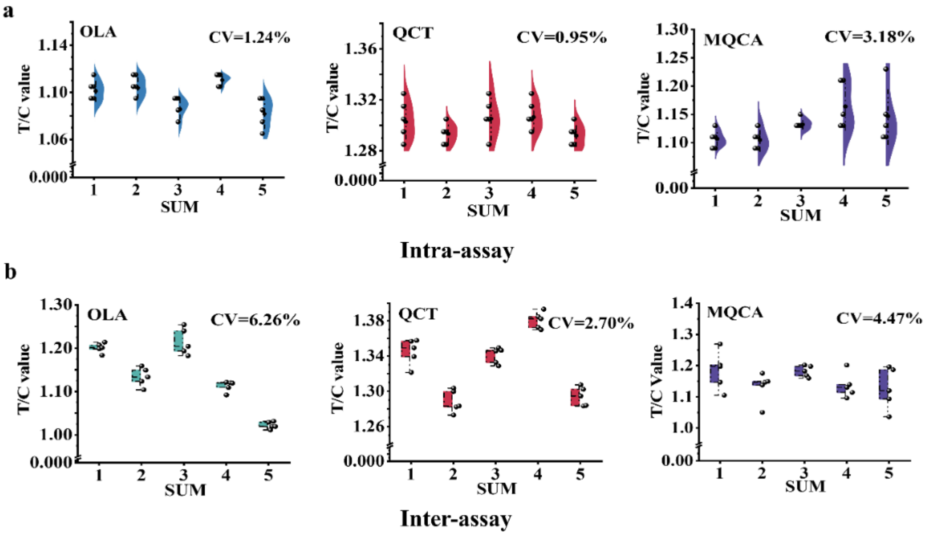

Figure S2. Intraassay and Interassay testing of EuNP–LFSBs. (a) Intraassay testing of EuNP–LFSBs (Intraassay precision 1–5 represents the same batch of 5 different test strips). (b)

Interassay testing of EuNP–LFSBs (Interassay precision 1–5 represents 5 different batches of test strips).

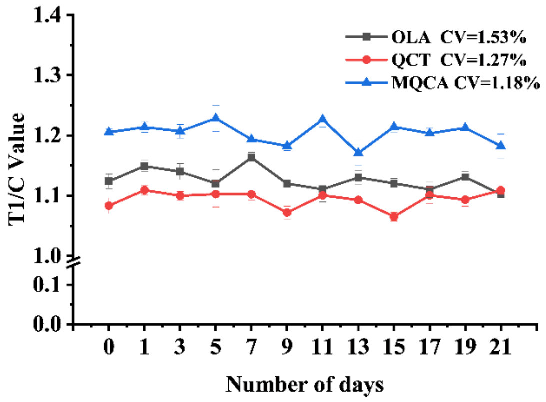

Figure S3. Stability results of the EuNP–LFSBs.

Optimization of the AuNP–LFSB system

The AuNP–LFSB system was optimized including the concentrations of three coating antigen and AuNP–mAb probes concentration, the mixed ratio of three AuNP–mAb probes, the best combination position of the three analytes, and the immunoreaction time.

In the study, the concentrations of three coating antigens and AuNP–mAb probes were optimized. The concentrations of OLA–OVA were 0.5, 1.0, 1.5, and 2.0 g/mL and the AuNP–OLA–mAb were 1.0, 1.5, 2.0, and 2.5 μg/mL. Similar parameter optimization was carried out for QCT and MQCA. Based on the T/C value, the concentration of OLA–OVA was 1.0 mg/mL and the AuNP–OLA–mAb was 1.5 μg/mL (Figure S4a). The concentration of QCT–OVA and EuNP–QCT–mAb was 1.5 mg/mL and 1.5 μg/mL (Figure S4b). The concentration of MQCA–BSA and EuNP–MQCA–mAb was 1.5 mg/mL and 2.0 μg/mL (Figure S4c).

The best mixed ratio of the three AuNP–mAb probes on the binding pad was another important factor affecting the sensitivity of the LFSB system. Thus, in this study, the three AuNP–mAb probes were optimized based on the volume ratio of 1:1:1 to 4:5:6. As shown in Figure S4d, according to the

T/C value and fluorescence intensity, the best mixed ratio of the AuNP–mAb probe was 1:1:2.

To obtain the best location of the binding area, the color rendering intensity of OLA, QCT, and MQCA was tested in three different binding areas. The result is shown in Figure S4e, OLA–OVA, QCT–OVA, and MQCA–BSA respectively located at the T1, T2, and T3 lines were the best binding areas (Figure S4e).

By detecting the signal intensity of the T and C lines at 1, 5, 10, 15, 20, 25, 30, 35, and 40 minutes, it could be seen from Figure S4f that the T/C value showed a stabilized and reached equilibrium with 10 minutes. To reduce the interference of non–specific background signals, thus, the optimal reaction time was selected as 10 min.

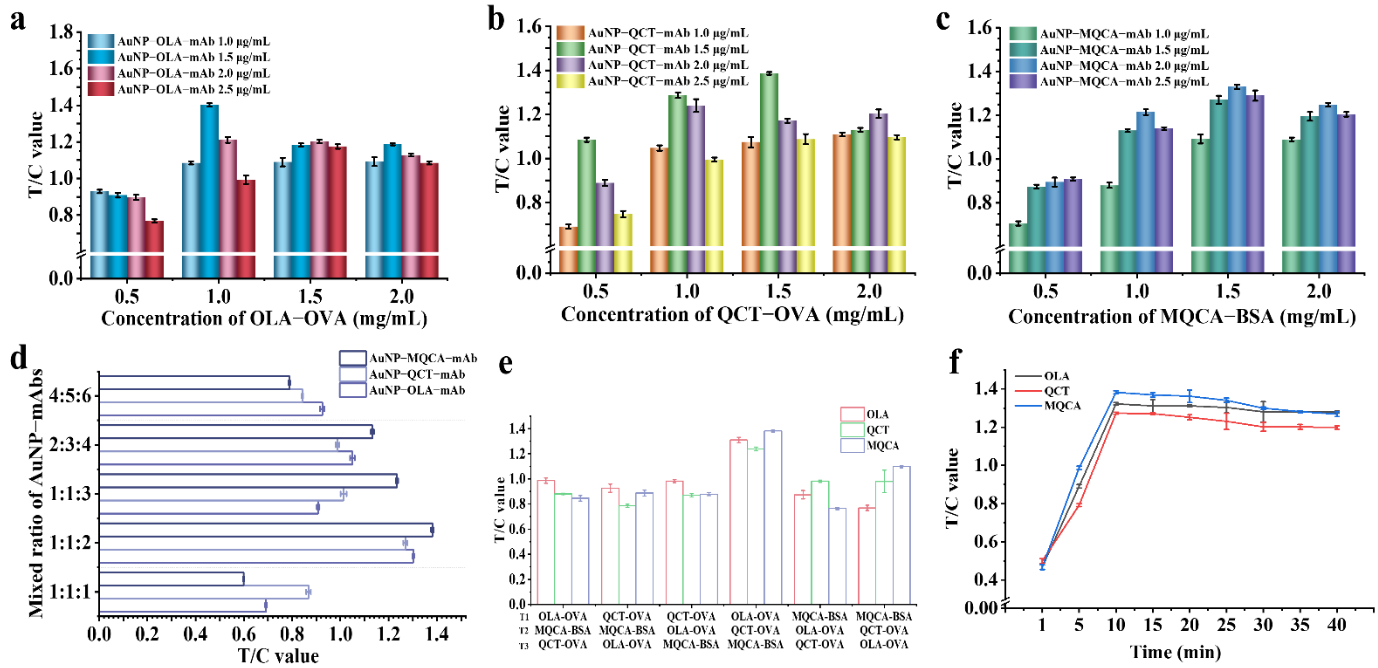

Figure S4. Optimization of the AuNP–LFSB system. (a) Influence of various OLA–OVA concentrations and AuNP–OLA–mAb concentrations on the AuNP–LFSBs. (b) Influence of various QCT–OVA concentrations and AuNP–QCT–mAb concentrations on the AuNP–LFSBs. (c) Influence of various MQCA–BSA concentrations and AuNP–MQCA–mAb concentrations on the AuNP–LFSBs. (d) The optimal results of the probe mixing ratios on the AuNP–LFSBs. (e) The result

of the position of the three encapsulated antigens on the AuNP–LFSBs. (f) The effect reaction time on AuNP–LFSBs.

Table S1. The results of EuNP–LFSBs and HPLC for actual samples.

Sample No.	EuNP–LFSBs (μg/kg)			HPLC (μg/kg)		
	QLA	QCT	MQCA	QLA	QCT	MQCA
9	—	1.451±0.260	—	ND	1.501±0.838	ND
29	—	2.131±0.980	—	ND	2.304±1.055	ND
46	1.430±0.103	—	—	1.493±0.451	ND	ND
63	—	1.513±0.415	—	ND	1.605±1.433	ND
169	1.518±0.280	—	—	1.619±0.211	ND	ND
252	—	1.401±2.521	2.247±1.531	ND	1.398±2.413	2.301±1.315
272	1.381±0.315	1.205±3.231	2.289±1.580	1.403±1.002	1.307±3.110	2.310±1.349
376	1.323±2.113	—	1.733±1.004	1.502±2.041	ND	1.934±1.421
420	1.260±0.518	—	2.235±1.104	1.114±1.023	ND	2.306±2.011
487	—	1.201±0.051	1.419±0.287	ND	1.250±1.211	1.514±0.983

ND: Not detected, "—": less than LOD.

ND/—:1–8, 10–28, 30–45, 47–62, 64–168, 170–251, 253–271, 273–375, 377–419, 421–486, 488–500.
